# Genetic Background Alters the Severity and Onset of Neuromuscular Disease Caused by the Loss of Ubiquitin-Specific Protease 14 (*Usp14*)

**DOI:** 10.1371/journal.pone.0084042

**Published:** 2013-12-16

**Authors:** Andrea G. Marshall, Jennifer A. Watson, Jada J. Hallengren, Brandon J. Walters, Lynn E. Dobrunz, Ludwig Francillon, Julie A. Wilson, Scott E. Phillips, Scott M. Wilson

**Affiliations:** Department of Neurobiology, Evelyn F. McKnight Brain Institute, Civitan International Research Center, University of Alabama at Birmingham, Birmingham, Alabama, United States of America; Columbia University, United States of America

## Abstract

In this study, we identified and characterized an *N*-ethyl-*N*-nitrosourea (ENU) induced mutation in *Usp14* (*nmf375*) that leads to adult-onset neurological disease. The *nmf375* mutation causes aberrant splicing of *Usp14* mRNA, resulting in a 95% reduction in USP14. We previously showed that loss of USP14 in *ataxia* (*ax* ^*J*^) mice results in reduced ubiquitin levels, motor endplate disease, Purkinje cell axonal dystrophy and decreased hippocampal paired pulse facilitation (PPF) during the first 4-6 weeks of life, and early postnatal lethality by two months of age. Although the loss of USP14 is comparable between the *nmf375* and *ax* ^*J*^ mice, the *nmf375* mice did not exhibit these *ax* ^*J*^ developmental abnormalities. However, by 12 weeks of age the *nmf375* mutants present with ubiquitin depletion and motor endplate disease, indicating a continual role for USP14-mediated regulation of ubiquitin pools and neuromuscular junction (NMJ) structure in adult mice. The observation that motor endplate disease was only seen after ubiquitin depletion suggests that the preservation of NMJ structure requires the stable maintenance of synaptic ubiquitin pools. Differences in genetic background were shown to affect ubiquitin expression and dramatically alter the phenotypes caused by USP14 deficiency.

## Introduction

Deficits in many different genes and pathways have been shown to contribute to neurological disease. In particular, the ubiquitin proteasome system (UPS) has been of great interest because alterations in protein turnover have been linked to chronic neurodegenerative disorders [1-3]. The presence of ubiquitin-positive inclusion bodies is a hallmark of many neurodegenerative disorders, including Alzheimer’s disease (AD), Parkinson’s disease (PD), Huntington disease (HD), and amyotrophic lateral sclerosis (ALS) [4]. Mutations in the deubiquitinating enzyme (DUB) UCHL1 have been linked to an autosomal dominant form of PD. Oxidative modification and down-regulation of UCHL1 are associated with both PD and AD [5–9]. Recently, mutations in a ubiquitin-like protein, UBQLN2, have been identified as causing dominant X-linked juvenile and adult-onset ALS and ALS/dementia [10]. In addition, mutations in the ubiquitin-activating gene *UBE1* have been identified in cases of X-linked infantile spinal muscular atrophy (XL-SMA), a devastating motor neuron disease that results in death in infancy [11]. These findings highlight the importance of ubiquitin signaling in maintaining proper neuronal function. 

Ubiquitination is a dynamic process that is utilized to regulate protein abundance. Through the activity of an E1 ubiquitin-activating enzyme, E2 ubiquitin-conjugating enzyme, and an E3 ubiquitin-ligase, ubiquitin is covalently linked to a lysine residue of a target protein [12]. Ubiquitinated proteins destined for degradation are shuttled to the proteasome, where the ubiquitin chain is disassembled through the activity of proteasomal DUBs before the protein is degraded. Proteasomal DUBs therefore prevent the degradation of ubiquitin and help to maintain the cellular pools of ubiquitin necessary for UPS function [13]. 

USP14 is a proteasomal DUB that trims ubiquitin chains conjugated onto proteins that are bound to the proteasome. We have previously used the *ax* ^*J*^ mouse model to demonstrate the importance of USP14 for neurodevelopment [14]. Loss of USP14 results in depletion of ubiquitin, deficits in hippocampal short-term synaptic plasticity, and Purkinje cell axonal dystrophy. Additionally, structural and functional deficits are observed at the NMJ of *ax* ^*J*^ mice, including neurofilament accumulation, terminal swelling, denervation, ultra-terminal sprouting, and defective synaptic transmission [15–17]. The NMJ deficits are attributed to ubiquitin depletion, because transgenic restoration of neuronal ubiquitin levels in the *ax* ^*J*^ mice improves the functional and structural deficits at the NMJs [16]. However, since the *ax* ^*J*^ mice succumb to early death, it is unclear if the activity of USP14 is required for the structure and function of NMJs throughout adult life. 

Here, we report a new mouse mutation, *nmf375*, which contains a recessive, ENU-induced mutation in *Usp14* and displays adult-onset motor endplate disease. Similar to the *ax* ^*J*^ mutation, the *nmf375* mutation led to an approximately 95% reduction in the levels of USP14. Although the *nmf375* mice displayed some motor and sensory deficits by 4-6 weeks of age, they did not have a significant reduction in monomeric ubiquitin levels, NMJ structural abnormalities, Purkinje cell axonal dystrophy or impaired hippocampal synaptic transmission that was characteristic of 4-6 week old *ax* ^*J*^ mice. The adult *nmf375* mice exhibited deteriorating motor performance, depletion of monomeric ubiquitin and motor endplate disease, indicating that USP14 is required for stable maintenance of adult motor endplates and ubiquitin pools. Backcrossing the *nmf375* and *ax* ^*J*^ mutations onto C57 and BALB/c, respectively, demonstrated that a genetic modifier(s) can alter the severity of neurological disease caused by USP14 deficiency. Collectively, our data indicate an ongoing requirement for USP14 at adult NMJs and show that genetic background has a major influence on the phenotypic expression of USP14 deficiency and the regulation of ubiquitin signaling in the nervous system.

## Methods

### Animals

The experiments described in this manuscript were approved by the University of Alabama at Birmingham Institutional Animal Care and Use Committee (IACUC). All research complied with the United States Animal Welfare Act and other Federal statutes and regulations relating to animals and experiments involving animals, and adhered to principles stated in the Guide for the Care and Use of Laboratory Animals, United States National Research Council. All reasonable efforts were made to minimize suffering of animals. The *nmf375* mutation was generated at the Jackson Laboratories Neuroscience Mutageneis Facility (NMF) as part of the neuromutagenesis initiative. C57BL/6J, BALB/c, *nmf375* and Usp14^*axJ*^ mice (Jackson laboratories, Bar Harbor, ME) have been maintained in our breeding colony at the University of Alabama at Birmingham, which is fully accredited by the Association for Assessment and Accreditation of Laboratory Animal Care International. Homozygous Usp14^*axJ*^, which we refer to as *ax* ^*J*^, and *nmf375* mice were maintained by intercrossing heterozygous siblings. The *ax* ^*J*^ and *nmf375* mice were genotyped using *D18MIT64F* (^5^’-TCA GAT TCA CTG CTA AGT CTT TTC-^3^’) and *D18MIT64R* (^5^’-AGC AAG AAA AGC AGG TGA GG-^3^’) primers. 

### Body mass and lifespan analysis

Body weights were collected from 4-, 8-, and 20-week-old C57BL/6J, BALB/c, *ax* ^*J*^, and *nmf375* mice. Weights and lifespan were determined for 4 animals per genotype. Values presented represent the average muscle mass ± SEM. The Student’s t-test was used to assess significance between C57BL/6J and *ax* ^*J*^ and BALB/c and *nmf375* at each time point.

#### NMJ immunostaining and confocal imaging

For whole mount staining, mice were processed as described previously [17]. Muscle bundles were incubated with antibodies to neurofilament heavy chain (Sigma, St. Louis, MO) and neurofilament medium chain (Santa Cruz Biotechnology, Santa Cruz, CA) in blocking buffer for 4 days at 4 °C. Following three 10 min washes in PBS + 1 % Triton X-100 (PBS-T), muscle bundles were incubated with secondary antibodies conjugated with Alexa Flour 488 dye (1:500, Invitrogen, Grand Island, NY) for 2 days at 4 °C. Muscle bundles were washed 5 times in PBS-T and once in PBS for 30 min at room temperature (RT) before mounting in 50% glycerol/PBS. Samples were imaged using a Leica TCS SP2 Laser Confocal (Leica, Wetzlar, Germany).

### Quantitative PCR (qPCR)

Total RNA was isolated using RNA-STAT60 (Tel-Test, Friendswood, TX) and was reverse transcribed using the GeneAmp Gold RNA PCR Reagent Kit (Applied Biosystems, Carlsbad, California). Real-time PCR reactions were set up in triplicate using TaqMan gene assays and amplified in an Applied Biosystems Step-1 instrument. Gene assay kits for individual assays were purchased from Applied Biosystems as previously described [17]. ΔΔCT curves were generated using 18S or GAPDH TaqMan gene assays as internal standards. qPCR results are shown as the relative quantity ± SD of three different amplifications from RNA reverse transcribed from three different mice. The Student’s t-test was conducted on relative quantity values for each group to determine their significance. 

### Protein Extracts

Brain and spinal cords were homogenized in modified RIPA buffer (10 mM Tris, pH 8; 1 mM EDTA; 0.5 mM EGTA; 1% Triton X-100; 0.1% sodium deoxycholate; 0.5% SDS; 140 mM NaCl). Homogenates were briefly sonicated, centrifuged at 16,100 x g at 4 °C for 15 min, and the supernatants were frozen at -80 °C.

### Immunoblotting

Proteins were resolved on either 10% Tris-glycine gels or 4–20% Tris-glycine NUPAGE gels (Invitrogen, Grand Island, NY) and transferred onto nitrocellulose or PVDF membranes. Antibodies against USP14 [18], ubiquitin (UAB Hybridoma Core, Birmingham, AL), αHA (12ca5, Santa Cruz Biotechnology), PSMC3 and PSMC4 (Aviva Systems Biology, San Diego, CA), 19S Proteasome α1 subunit (Santa Cruz Biotechnology), and β-tubulin Ab (Developmental Hybridoma Core, Iowa City, IA) were diluted in PBS containing 2% (w/v) BSA and 0.1% NP-40. Primary antibodies were detected using an anti-mouse or anti-rabbit HRP-conjugated secondary antibody (1:5000; Southern Biotechnology Associates, Birmingham, AL) and Supersignal West Pico (Thermo Fisher Scientific, Rockford, IL) reagents.

### Quantitation of immunoblots

Blots were scanned using a Hewlett-Packard Scanjet 3970 and quantified using ImageJ software (NIH, Bethesda, MD). Each value represents the average ± SEM from at least three blots using at least three different animals of each genotype. Statistics were performed using the Student’s t-test.

### Proteasome fractionation and DUB activity assay

Brains were homogenized in HR buffer (50 mM Tris, pH 7.4; 5 mM MgCl_2_; 250 mM sucrose; 1 mM dithiothreitol; 2 mM ATP) on ice. Large cellular debris were pelleted by centrifugation at 10,000 x g for 10 min at 4 °C. The supernatant was centrifuged at 100,000 x g for 1 h at 4 °C, followed by a 100,000 x g spin for 5 hr at 4 °C to pellet proteasomes. Proteasomes were resuspended in HR buffer and stored at -80 °C. For DUB labeling assays, 20 μg of proteasomes were incubated in HA-Ubiquitin-Vinyl Methyl Ester (HA-UB-VME) (Enzo Life Sciences, Farmingdale, NY) for 30 min at RT. Reactions were terminated with Laemmli sample buffer, boiled for 5 min and used for immunoblot analysis and probed for the HA epitope. 

### Proteasome activity assay

The trypsin-like activity of the 20S proteasome was measured using the substrate Boc-LRR-AMC (Enzo Life Sciences). The substrate was added to 2 μg of proteasomes in a 100 μL total reaction volume (20 mM Tris-HCl, pH 7.5; 1mM EDTA; 1 mM NaN_3_; 1 mM dithiothreitol) at 100 μM concentration. Reactions were performed in triplicate. Fluorescence activity was measured at 37 °C at an excitation wavelength of 360 nm and an emission wavelength of 460 nm on a SpectraMax M3 microplate reader (Molecular Devices, Sunnyvale, CA). Intensity was recorded at 5 min intervals for 1 h. The proteasome inhibitor MG132 (Sigma) was added at a final concentration of 50 μM as a negative control for each reaction. Graphs shown are the average autofluorescence units ± SEM at 60 min for each genotype. Statistics were performed using the Student’s t-test.

#### Open field analysis

General locomotor activity of 4-6 week-old animals was examined by Open Field Activity System. Animals were habituated to the behavior room 30 min prior to analysis (n = 4 per genotype). Animal movement in a 43.2 x 43.2 x 30.5 cm box was tracked for 15 min. The first 5 min were not analyzed to account for habituation to the open field apparatus. Analysis was performed using the distance traveled in the center and the total distance traveled for each genotype. 

### Behavioral Assays

Motor and sensory function was assayed at 4-6 weeks of age (n≥4). These measures were repeated when the animals were 10-12 weeks of age. Before each trial, animals were habituated to the testing room for 30 minutes. Student t-test was performed on grip strength and von Frey data, and a two-way ANOVA was utilized to determine significance of rotarod assays. Motor coordination and balance was tested by placing mice on an accelerating rotarod (ENV-575, Med Associates, St. Albans, VT). The rotarod started at 3 rpm and accelerated to 30 rpm over a 5 minute period and latency to fall was recorded. Each mouse performed 3 trials separated by an hour. A Grip Strength System (San Diego Instruments, San Diego, CA) was used to assay mouse forelimb grip strength. The maximum amount of force generated from forelimbs was recorded. Each trial consisted of 12 repetitions of the assay with the two highest and two lowest data points dropped from final analysis. A von Frey analysis was performed to determine tactile sensation. Animals were habituated to an open gridded floor chamber for 5 minutes. A series of 10 von Frey fibers varying from 0.4 g to 60 g of force (Ugo Basile, Comerio, Italy) was applied from below the wire mesh chamber in ascending order beginning with the smallest fiber. Fiber was applied to the central region of the plantar surface. The hind paw withdrawal threshold was determined by Dixon’s formula. 

### Calbindin staining

Brains were dissected, cut sagittally and placed in 50 mL Methacarn fix (60% methanol, 30% chloroform, 10% glacial acetic acid) overnight at 4 °C. The tissue was then dehydrated in 70% ethanol overnight at 4 °C before paraffin embedding. Sagittal sections (7-10 μm) were cut from the midline, mounted onto slides, and processed as previously described [17]. Slides were blocked in PBS containing 1% (w/v) BSA, 0.2% (w/v) dried nonfat milk, and 0.1% Triton X-100 (PBS-BB) for 30 min at RT. Slides were incubated overnight with an anti-calbindin antibody (Swant, Switzerland) at 4 °C. After washing 3 times in PBS for 5 min at RT, Alexa 568 secondary antibody (Invitrogen) in PBS-BB was then applied for 1 h at RT. Slides were washed 3 times in PBS for 5 min before mounting with VectaShield with DAPI (Vector Laboratories Inc., Burlingame, CA) to stain nuclei.

### Sequencing of the *Usp14* open reading frame

RNA was isolated from brain utilizing RNA-STAT60. First strand cDNA synthesis was performed with Superscript III First-Strand Synthesis System (Invitrogen). Primer sets were designed to isolate the *Usp14* transcript (^5^’-GTT CAG TGT ATT CGT TCT GTG CC-^3^’ and ^5^’-TTT CCT TTC CTT TGG TGA CCT C-^3^’). Transcripts were cloned with the Perfectly Blunt Cloning Kit (EDM, Billerica, MA), and DNA sequences were determined by the UAB Heflin Center for Genomic Science. 

### Exon-specific sequencing of *Usp14*

Exons 7-11 of *Usp14* were amplified from isolated tail DNA using sequence specific primers (Exon 7-f ^5^’-TGG AAC TCA CTT TGT AGA CCA GGC-^3^’; exon 7-r ^5^’-AGG ACC AGC ACC AGA GGT TGT C-^3^’; exon 8-f ^5^’-CCA AGG GTA GGA TTG TAG GTG TGC-^3^’; exon 8-r ^5^’-AGG GAA GCA ACC AGG TCA GC-^3^’; exon 9-f ^5^’-TGT CCC CAT CTT TGT CAC AGG-^3^’; exon 9-r ^5^’-CAG TGC CAA TCA GCC AAC TAT G-^3^’; exon 10-f ^5^’ -TGA TAA AGT GGG TAG GCA AAT GAA C-^3^’; exon 10-r ^5^’-AGG AAT GCT GTA TGC TTG GGG-^3^’; exon 11-f ^5^’-TTG TGT CCT TGA CCC CCT CAG-^3^’; exon 11-r ^5^’-AGC CAC CTC CTT CCC CTT GAA C-^3^’). Amplicons were sequenced by the UAB Heflin Center for Genomic Science. DNA was isolated from tail clips from congenic BALB/c, C57BL/6J, *nmf375*, and C3H/HeJ mouse lines maintained in our animal colony. The UAB Transgenic Core kindly provided the DNA from FVB and DBA inbred mice. 

### Acute Brain Slice Preparation

BALB/c or *nmf375* mice (4-6-weeks-old) were deeply anesthetized with isoflurane, decapitated, and their brains were removed rapidly. A vibrating microtome (VT1000S; Leica, Bannockburn, IL) was used to cut 400 μm thick coronal slices in ice-cold dissecting solution containing the following (in mM): 120 NaCl, 3.5 KCl, 0.7 CaCl_2_, 4.0 MgCl_2_, 1.25 NaH_2_PO_4_, 26 NaHCO_3_, and 10 glucose, bubbled with 95% O_2_-5% CO_2_, pH 7.35–7.45. Brain slices were allowed to recovered for at least 1 h prior to recording in recording solution containing the following (in mM): 120 NaCl, 3.5 KCl, 2.5 CaCl_2_, 1.3 MgCl_2_, 1.25 NaH_2_PO_4_, 26 NaHCO_3_, and 10 glucose, bubbled with 95% O_2_-5% CO_2_, pH 7.35–7.45.

### Field Recordings

Experiments were performed in a submersion recording chamber at 28-30 °C. Brain slices were perfused with extracellular recording solution described above, with the addition of 100 μM APV ((2R)-amino-5-phosphonovaleric acid) (Sigma) to block NMDAR-mediated plasticity. Extracellular field potentials were elicited with 100 μs duration pulses via tungsten microelectrodes placed in the stratum radiatum. Dendritic field excitatory post-synaptic potentials (fEPSPs) were recorded using glass micropipettes (2–4 MΩ) filled with extracellular solution placed in the stratum radiatum. Experimental stimuli were set to an intensity that evoked a fEPSP that had a left slope of ~50% of the maximum fEPSP needed for population spike generation. After a stable baseline recording was obtained with 0.1 Hz stimulation for 15 min, paired pulse facilitation (PPF) was tested at 50 ms, 100 ms, 150 ms, 200 ms, and 500 ms interstimulus-intervals. The paired pulse ratio (PPR) was obtained by dividing the slope of the second facilitated pulse by the slope of the first pulse. Experiments for a single animal were averaged before averaging multiple animals. 

### Muscle fiber analysis

Fiber analysis was conducted as previously described [17]. For cross-sectional measurements, 25-70 random type I and type II myofibers from each animal were manually traced along their laminin-stained border using ImageJ software. Statistics were performed using the Student’s t-test.

## Results

### Genetic mapping of the *nmf375* mutation

**Figure 1:**
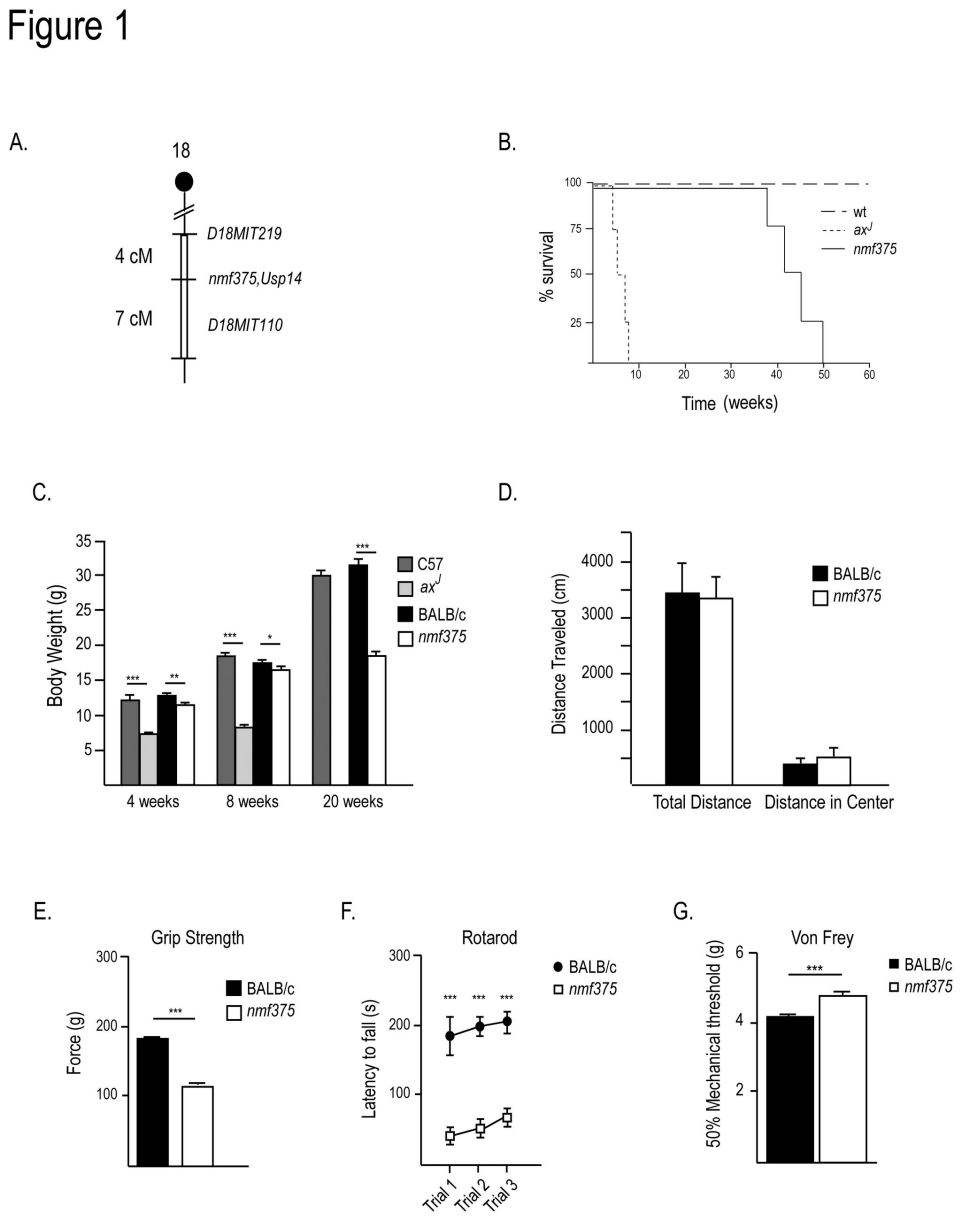
*Usp14* maps to the *nmf375* critical region. *A*, Meiotic and physical linkage map of the *nmf375* critical region. Filled circle denotes the centromere, open box represents critical region. *B*, Survival curves of wt (C57BL/6J and BALB/c), *ax* ^*J*^, and *nmf375* mice. *C*, Body weights from 4- to 20-week-old C57BL/6J, BALB/c, *ax* ^*J*^, and *nmf375* mice. *D*, Open field Analysis. ***E***, Measurements of forelimb grip strength. *F*, Rotarod Analysis. ***G***, Von Frey assay for tactile sensitivity. *D*-*G*, mice were 4-6 weeks of age. *p-value <0.05, **p-value <0.01, ***p-value <0.001. (n = 4-6 mice per genotype). Data were analyzed by Student’s t-test and are shown as mean ±SEM.

 The recessive neurological mutation *nmf375* was generated through ENU mutagenesis at the Jackson Laboratory and displayed hind limb muscle weakness and muscle spasms. Genetic linkage analysis from the Jackson Laboratory localized the *nmf375* mutation to a 11 cM region on proximal chromosome 18 (Fig. 1A). Examination of the genes within this region revealed *Usp14* as an excellent candidate, as mutation of this gene in the neurodevelopmental *ax* ^*J*^ mice results in hind limb weakness, reduced muscle mass and postnatal lethality by 2 months of age [14]. To determine if the *nmf375* mutation was within *Usp14*, we performed a complementation analysis by mating heterozygous *nmf375/+* mice with heterozygous *ax* ^*J*^ */+* mice. Approximately 25% of the resulting offspring exhibited the *ax* ^*J*^ phenotype, indicating that the *nmf375* allele was unable to complement the *ax* ^*J*^ allele and, therefore the *nmf375* mutation resides within *Usp14*.

### Phenotypic Characterization of *nmf375* Mice

*nmf375* mutants displayed a distinctly different phenotype than the *ax* ^*J*^ mutants. *nmf375* mutants have an intermediate lifespan compared to *ax* ^*J*^ mutants and controls (Fig. 1B). While all *ax* ^*J*^ mice died by 2 months of age, the *nmf375* mice survived beyond 8 months of age and died by 50 weeks. Although the *nmf375* mutants were significantly smaller than controls by 4 weeks of age, they continued to increase in mass over time, which is in contrast to what was observed in the *ax* ^*J*^ mice (Fig. 1C). Despite being smaller, baseline locomotion of *nmf375* mutants was not altered from controls in open field analysis at 4-6 weeks of age (Fig. 1D), a time point where the *ax* ^*J*^ mice exhibited severe motor abnormalities [19]. While overall motor activity was not altered between *nmf375* mice and controls, *nmf375* mice displayed subtle changes in peripheral nervous system function. Behavioral assays revealed muscle weakness, impaired motor coordination, and decreased tactile sensation (Fig.1E, F, G). Together, these data demonstrate that *nmf375* mice have significantly milder peripheral nervous system deficits than the *ax* ^*J*^ mice, but reaffirm the role of USP14 in the normal development and function of motor and sensory circuits. 

### Identification of the *nmf375* mutation and analysis of *Usp14* gene products from the *nmf375* mice

**Figure 2:**
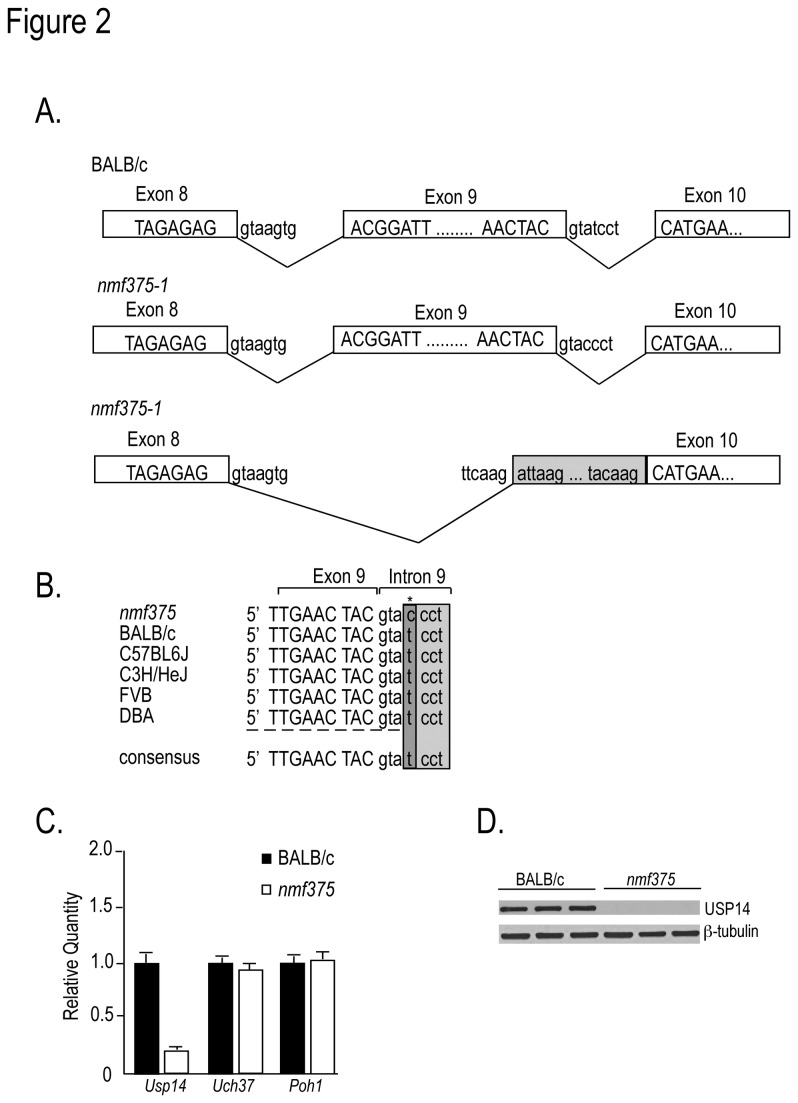
Characterization of *Usp14* in the *nmf375* mice. *A*, Schematic diagram of *Usp14* cDNA species amplified. Open box indicates exonic sequence, lower case letters denote intronic sequence, grey box indicates intronic sequence maintained in mature transcript, angled lines represent splicing pattern. *B*, Genomic DNA sequence of *Usp14* exon 9/intron 9 border from *nmf375*, BALB/c, C57BL/6J, C3H/HeJ, FVB, and DBA mice. Capital letters denote exonic sequence; lower case letters and grey box denotes intronic sequence. Asterisk and dark grey box denotes mutation. *C*, Representative qPCR of *Usp14*, *Uch37, Poh1* mRNA from spinal cord extracts of 4-6 week old BALB/c and *nmf375* mice (n = 3 per genotype). Data were analyzed by Student’s t-test and are shown as mean ±SEM. *D*, Representative immunoblot of USP14 from spinal cord extracts from 4-6 week old BALB/c and *nmf375* mice (n = 3 per genotype). Blots were also probed for β-tubulin as a loading control.

To identify the location of the *nmf375* mutation, the cDNA sequence of the *Usp14* gene was determined from *nmf375* and BALB/c control mice. Sequence analysis revealed that the *nmf375* mice produced two *Usp14* transcripts: one indistinguishable from BALB/c controls and a second in which exon 9 was excluded and a portion of intron 9 was retained in the mature mRNA (Fig. 2A). Under normal conditions, exon 9 does not undergo alternative splicing [14]. The presence of this aberrant *Usp14* transcript suggested that the *nmf375* mutation might lie between exons 8-10 of *Usp14* and affect pre-mRNA splicing of *Usp14*. 

ENU-mutagenesis is known to cause point mutations, some of which have been shown to affect pre-mRNA processing [20–23]. Comparison of *Usp14* genomic sequence between BALB/c and *nmf375* mice revealed a thymine to cytosine (TC) base transition in position 4 of the splice-site donor region of intron 9 in the *nmf375* mutants (Fig. 2C). To confirm that this TC transition was not a naturally occurring single nucleotide polymorphism (SNP), we sequenced the exon 9/intron 9 region of *Usp14* from several inbred mouse strains (C57BL/6J, C3H/HeJ, FVB, and DBA) and found conservation of the T residue at position 4 of the splice site donor region in all of the strains analyzed (Fig. 2B). To confirm our sequencing data, we searched for known SNPs within the intron 9 region using the Mouse Genomes Informatics (http://www.informatics.jax.org/) and the Mouse Genomes Project (http://www.sanger.ac.uk/resources/mouse/genomes/) SNP databases. No SNPs have been reported in the splice site donor region of intron 9 of *Usp14*.

The *ax* ^*J*^ allele is a hypomorphic allele that decreases *Usp14* mRNA stability due to the retention of an intracisternal-A Particle in the fully processed mRNA [14]. To determine if the *nmf375* mutation also affects *Usp14* mRNA stability, we performed qPCR analysis to measure levels of *Usp14* transcripts in 4-6 week old BALB/c and *nmf375* mice. The qPCR primers used to examine *Usp14* transcript levels amplify all *Usp14* cDNAs and do not distinguish between the splice-variants. The qPCR results showed an 80% reduction in *Usp14* mRNA in *nmf375* mutants compared to controls (Fig. 2C). This decrease in *Usp14* expression did not result altered expression of the other ubiquitin proteasomal DUBs *Poh1* and *Uch37* (Fig. 2C). To determine the effect of decreased *Usp14* mRNA on USP14 protein expression, immunoblot analysis was used to measure the steady state level of USP14 in *nmf375* mice compared to controls. USP14 was not readily detectable in *nmf375* spinal cord extracts using the USP14 polyclonal antisera (Fig. 2E). This effect on USP14 expression was similar to what we have previously described for the *ax* ^*J*^ mice [14]. Although *Usp14* transcripts could be detected in the *nmf375* mice, it appears that most of these transcripts did not lead to the production of a functional protein. In addition, no truncated forms of USP14 were detected using our polyclonal antisera or antisera that was directed against epitopes in the first 300 amino acids of USP14 [14]. Together, these data indicate that the *nmf375* mutation is a hypomorphic allele of *Usp14*. Surprisingly, despite the dramatic reduction in USP14, *nmf375* mutants do not exhibit the severe, early-onset, neurological disease that we have previously reported for the *ax* ^*J*^ mice that are deficient for USP14 [14]. 

### Examination of ubiquitin homeostasis in the *nmf375* mice

**Figure 3:**
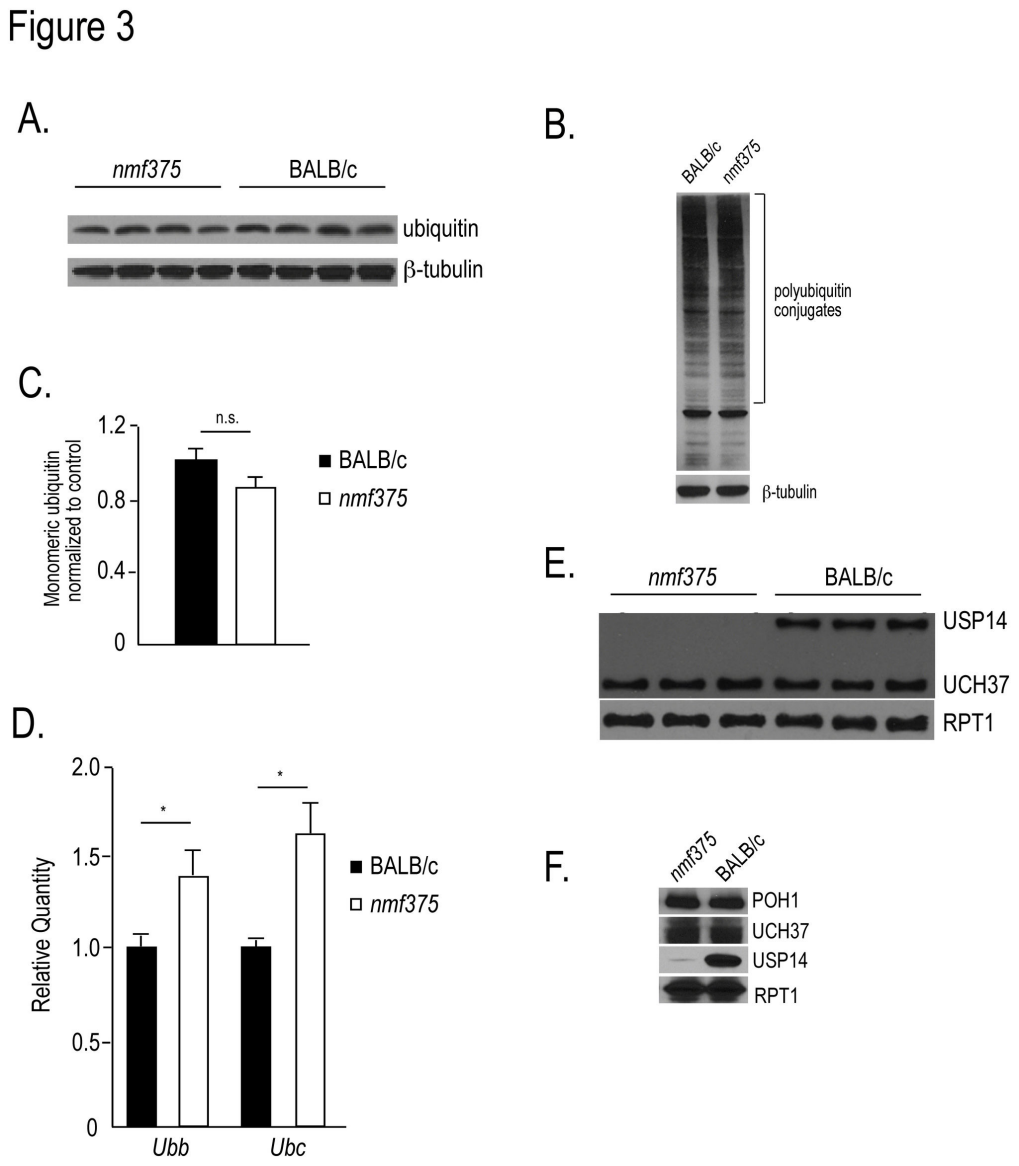
Analysis of ubiquitin expression in *nmf375* mutants at 4-6 weeks of age. *A*, Representative immunoblot of monomeric ubiquitin from spinal cord extracts BALB/c and *nmf375* mice (n = 4). *B*, Representative immunoblot of polyubiquitin conjugates from BALB/c and *nmf375* spinal cord extracts. *C*, Quantitation of monomeric ubiquitin levels from A (p = 0.12). *D*, Representative qPCR of ubiquitin *Ubb* and *Ubc* mRNA from spinal cord extracts of BALB/c and *nmf375* mice (n=3 per genotype); *p-value <0.01. *E*, Active DUBs in proteasome extracts isolated from the brains of BALB/c and *nmf375* mice were labeled with HA-UB-VME and probed with an anti-HA antibody. Blots were also probed for the α1 subunit (RPT1) of the 19S proteasome as a loading control (n = 3 per genotype). *F*, Representative immunoblot of proteasome extracts from BALB/c and *nmf375* mice probed for POH1, UCH37, USP14 and RPT1. Data were analyzed by Student’s t-test and are shown as mean ±SEM.

 USP14 is necessary for maintaining cellular ubiquitin pools. In yeast, loss of the USP14 homolog Ubp6 results in increased ubiquitin turnover by the proteasome [24]. In mice, loss of USP14 results in a rundown of ubiquitin levels as well as developmental abnormalities at the NMJ and in the CNS, suggesting that USP14 is required to maintain ubiquitin pools necessary for nervous system development [16,18]. Therefore, we examined the effect of the *nmf375* mutation on neuronal ubiquitin homeostasis. The lack of severe neurological disease within the first 4-6 weeks of life suggested that *nmf375* mutants were able to regulate ubiquitin levels even in the absence of USP14. In support of this idea, immunoblot analysis of spinal cord extracts from 4 to 5 week-old *nmf375* mutants did not reveal a significant difference in the level of free or conjugated ubiquitin compared to controls (Fig. 3A, B, C). However, qPCR analysis of the ubiquitin genes *Ubb* and *Ubc*, which are upregulated under conditions of cellular stress [25], revealed significant increases in both genes in *nmf375* mice compared to controls (Fig. 3D). Although there was no significant reduction in ubiquitin protein levels in the *nmf375* mice at this time point, the increased ubiquitin transcription likely indicated that there was increased degradation of ubiquitin by the USP14 deficient proteasomes (Figure 3C). 

 To determine if an increase in the activity of other proteasomal DUBs may help to stabilize ubiquitin pools in the *nmf375* mice, we used an HA-UB-VME probe to identify active DUBs in proteasome extracts isolated from *nmf375* and control brains [18]. As expected, HA-labeled USP14 and UCH37 were detected in proteasome fractions from BALB/c mice, while *nmf375* fractions only contained labeled UCH37 (Fig. 3E). No significant differences were observed in the amount of HA-labeled UCH37 in BALB/c and *nmf375* proteasome fractions. In addition, when we examined the amount of POH1, UCH37 and USP14 in proteasome fractions from brains of BALB/c and *nmf375* mice, no significant changes were observed in the levels of POH1 or UCH37 in the *nmf375* mice (Fig. 3F). As expected, the level of USP14 was greatly reduced in the proteasome fraction isolated from *nmf375* mice as compared to controls.

### Examination of motor endplates in the *nmf375* mice

**Figure 4:**
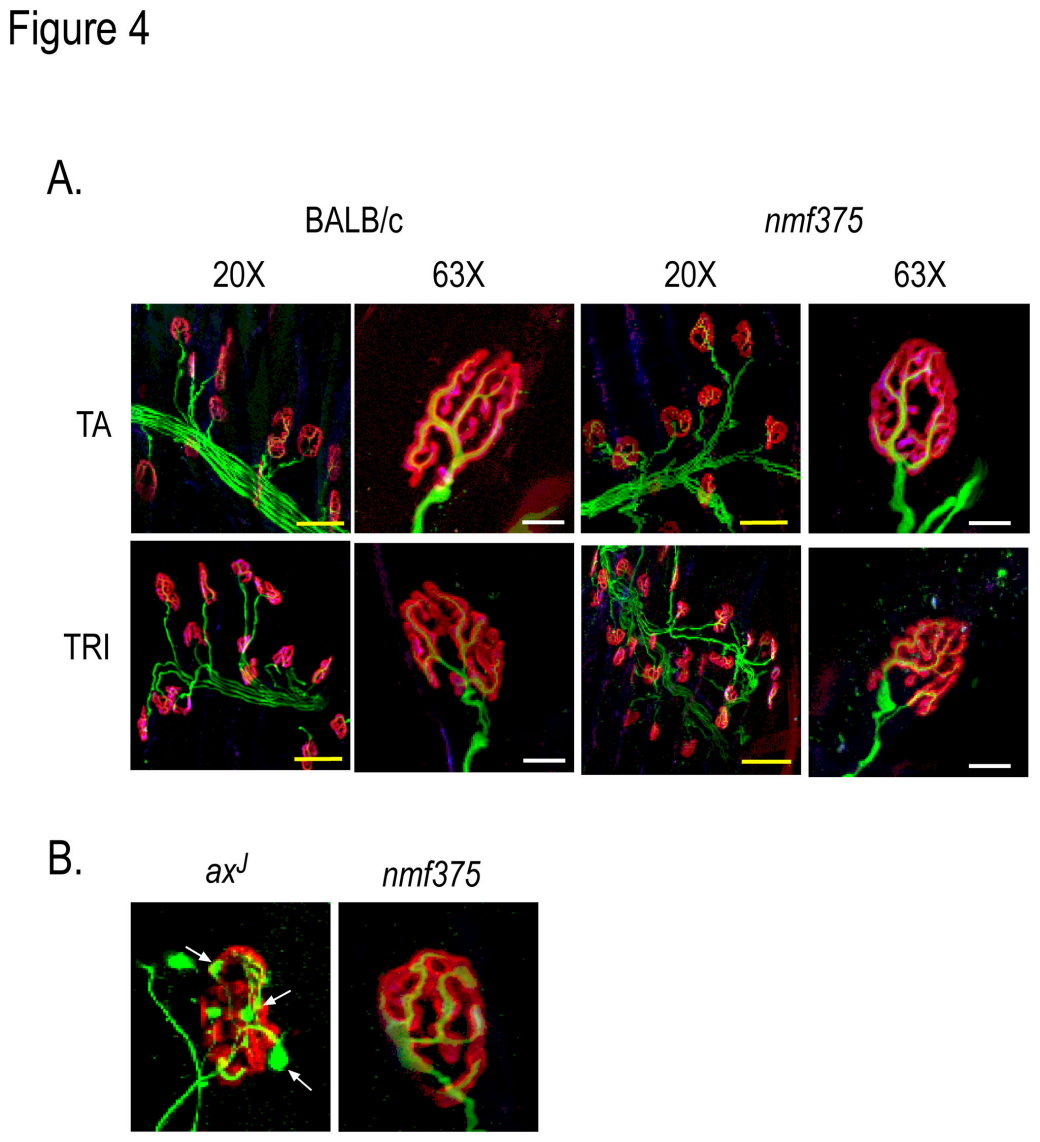
Structural analysis of NMJs from 4-6 week-old BALB/c and *nmf375* mice. *A*, The NMJs of the tibialis anterior (TA) and triceps brachii (TRI) muscles from *nmf375* and BALB/c mice were stained for neurofilament (green) and α-bungarotoxin (red) and imaged using confocal microscopy. Left: 20X magnification, scale bar 50 μm; Right: 63X magnification, scale bar 20 μm. *B*, Comparison of NMJs from TA muscles of *ax* ^*J*^ and *nmf375* mice stained as in A. Note the NMJs from *ax* ^*J*^ muscle contain terminal swellings (white arrows).

One of the consequences of the loss of USP14 in *ax* ^*J*^ mice is the accumulation of neurofilaments at motor neuron endplates, which correlates with decreased levels of ubiquitin [16,17]. To examine motor endplate structure, we performed whole-mount immunostaining of tibialis anterior (TA) and triceps brachii (TRI) muscles from 4-6 week-old BALB/c and *nmf375* animals (Figure 4). Motor axons and presynaptic terminals were visualized using antibodies against the heavy- and medium- neurofilament subunits, and motor endplate acetylcholine receptors (AChR) were visualized using rhodamine-conjugated α-bungarotoxin. As predicted, NMJs from the *nmf375* mutants were structurally indistinguishable from wild type controls. The staining of motor axons tightly overlaid the staining observed for the AChRs (Figure 4A), and no swellings or accumulations of neurofilaments were seen in terminals of *nmf375* mutants. In contrast, as we have previously shown [17], terminals from 4-week-old *ax* ^*J*^ mutants showed large accumulations of neurofilaments (Figure 4B). These data support our previous finding that neuronal ubiquitin levels are critical for the proper structural development of the NMJ [16].

### Examination of AChR subunit expression and muscle fiber size

**Figure 5:**
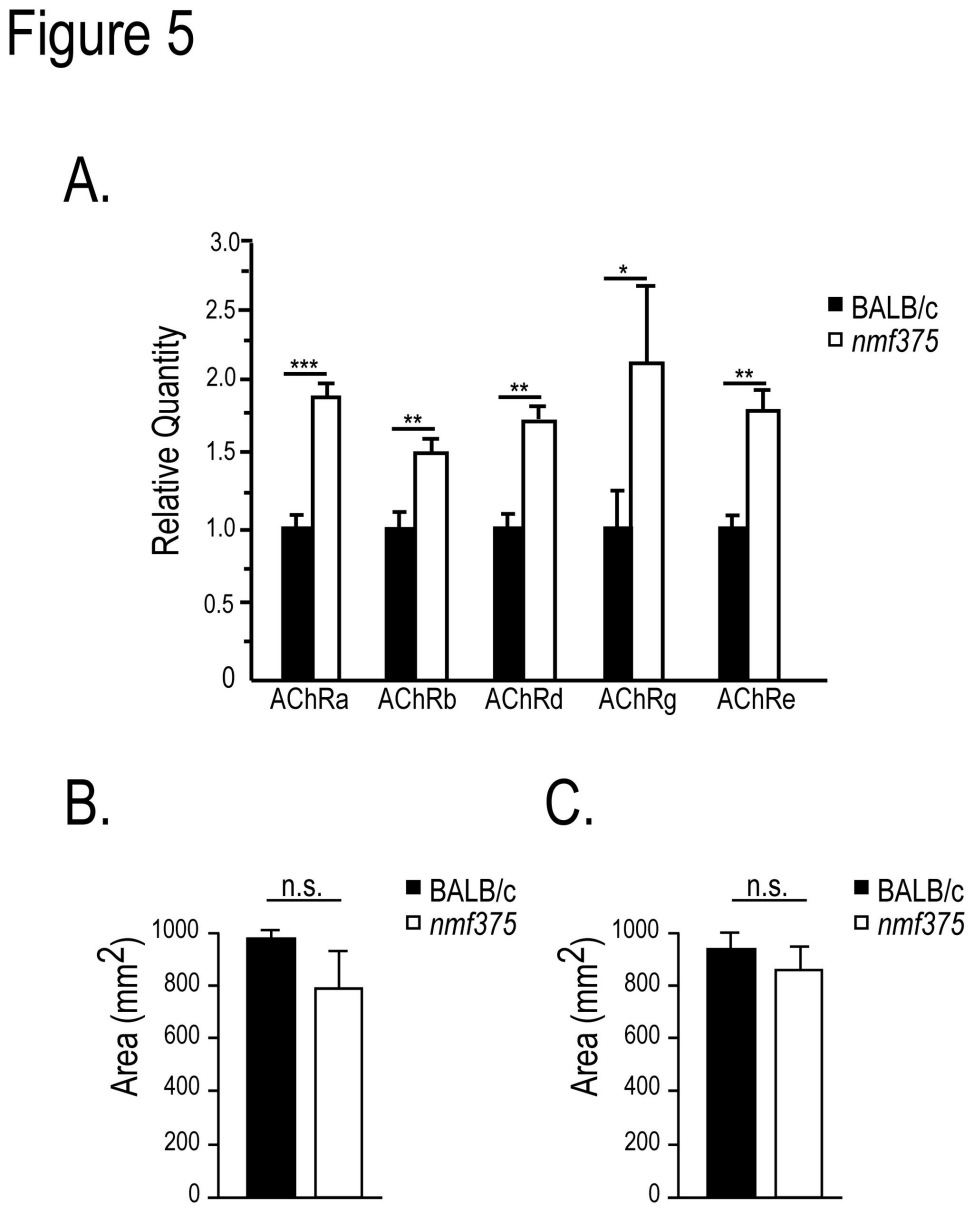
Examination of muscle AChR subunit expression and fiber type cross-sectional area of gastrocnemius muscle from 4-6 week-old BALB/c and *nmf375* mice. *A*, qPCR of α, β, δ, γ, and ε AChR subunit mRNAs from gastrocnemius muscles; *p-value <0.05; **p-value <0.01, ***p-value <0.001. n = 3 per genotype. *B*-*C*, Quantitation of type I (B) and type II (C) fiber cross-sectional area from gastrocnemius muscles (n = 3 per genotype, 25-60 fibers per animal). Data were analyzed by Student’s t-test and are shown as mean ±SEM.

 Although the *nmf375* mutants are visibly smaller than controls and exhibit peripheral nervous system dysfunction (Fig. 1E, F), the NMJs of 4-6 week-old *nmf375* mice appear to be resistant to the structural alterations caused by the loss of USP14. We therefore predicted that the functional deficits caused by loss of USP14 contribute to the reduced muscle development seen in the *nmf375* mice. Loss of neurotransmission at the NMJ due to injury or toxins has been shown to increase expression of muscle AChR subunits [26,27]. In *ax* ^*J*^ mice, the loss of USP14 results in reduced synaptic transmission and a compensatory increase in muscle AChR expression [14,17]. As an indirect measure of synaptic transmission at the NMJ, qPCR analysis of RNA isolated from the gastrocnemius muscle of 4-6 week-old *nmf375* and control mice was used to monitor the expression of AChR subunit expression (Fig. 5A). We observed a significant upregulation in expression of all five AChR subunits in *nmf375* mice compared to controls (Fig. 5A), which is consistent with altered synaptic transmission at the NMJ and provides evidence that the *nmf375* mutants are better able to tolerate the loss of USP14 [17]. 

 Loss of neurotransmission due to nerve crush injury causes a decrease in both type I and type II muscle fiber diameter and grouping of fiber types [28]. Thus, an alternative method to indirectly assess neuronal function at the NMJ is to measure type I and type II muscle fiber volume. When we examined muscle fibers from 4-week-old *ax* ^*J*^ mutants, which have altered function at the NMJ [14], there was a significant reduction in the cross-sectional area of type I and type II fibers from the gastrocnemius muscles, suggesting insufficient motor neuron input for proper muscle development and growth [17]. To determine if the *nmf375* mice displayed a similar defect in muscle development, gastrocnemius muscles from 4-6 week-old *nmf375* and control mice were sectioned and stained for type I fibers. In contrast to what we previously reported for the *ax* ^*J*^ mice [17], the cross-sectional area of muscles fibers seen in *nmf375* mice was similar to that of controls (Figure 5B, C). 

### Examination of Purkinje cell axonal morphology

The original report describing the *ax* ^*J*^ mutants detected swellings in the axons of Purkinje cells as early as 3 weeks of age [29]. Although our previous studies have shown that these swellings are not the major cause of the motor deficits seen in the *ax* ^*J*^ mice [19], they do contribute to the altered gait observed in these mice. To examine if the *nmf375* mutants displayed Purkinje cell axonal pathology, whole-brain sagittal slices were immunostained with antibodies against calbindin to label the Purkinje cell body and processes. As controls, whole-brain sagittal slices from *ax* ^*J*^ and C57 controls were also immunostained and used for comparison. While large Purkinje cell axonal swellings were observed throughout the *ax* ^*J*^ sections [19], axons from 4-6 week-old *nmf375* mutants did not exhibit these swellings (data not shown). These data suggest that *nmf375* mutants are resistant to changes in Purkinje cell axonal pathology caused by the loss of USP14.

### Investigation of hippocampal short-term synaptic plasticity at Schaffer Collateral synapses in *nmf375* mice

**Figure 6:**
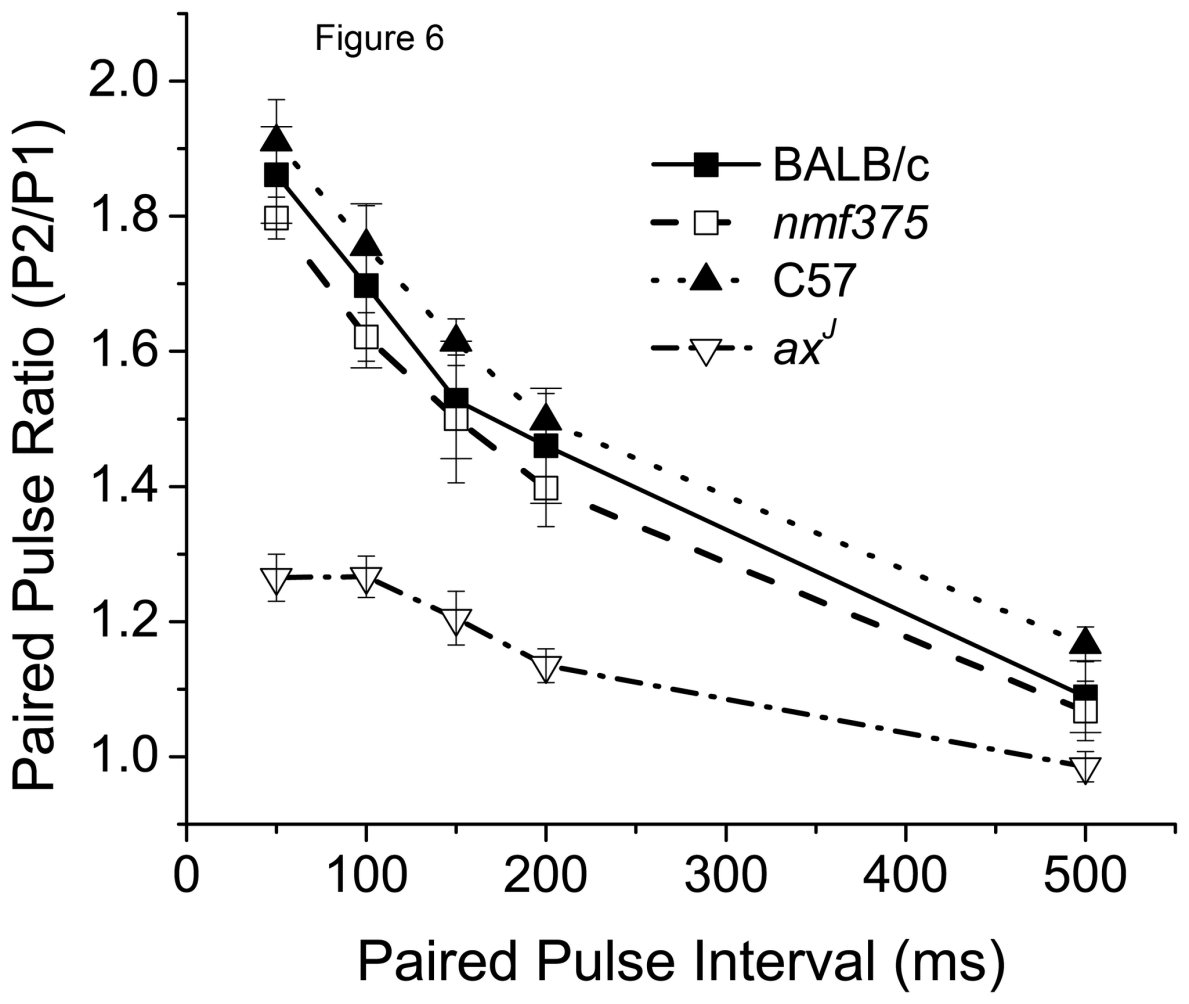
Hippocampal paired pulse facilitation (PPF) in BALB/c, *nmf375*, C57, and *ax* ^*J*^ mice at 4-5 weeks of age. Dendritic field EPSPs were measured in the CA1 region of the hippocampus in response to extracellular stimulation of Schaffer collateral axons. Two stimuli, spaced at the time intervals indicated, were given at 0.1Hz, and the paired pulse ratio at each interval was obtained by dividing the initial slope of the second pulse by the initial slope of the first pulse (n= 3-4 animals per group).

 The progressive hind limb weakness in *nmf375* mice, paired with the upregulation of AChR subunits at the NMJ, suggests reduced synaptic transmission at the NMJ in these mice. In order to determine if synaptic transmission is altered at central synapses, we examined paired-pulse facilitation (PPF) in the hippocampus (Fig. 6). Paired stimuli with a range of interpulse intervals were applied to Schaffer collateral axons, and excitatory post-synaptic potentials were measured in the dendritic field of area CA1 in acute slices from 4-6-week-old mice. In control animals, the response to the second stimulus was greater than the response to the first stimulus, demonstrating short-term facilitation at this synapse (Fig. 6). As we have previously shown [14], PPF was reduced across a wide range of interpulse intervals in the *ax* ^*J*^ mice compared to C57 controls (Fig. 6). In contrast, the PPF observed in the *nmf375* mice was indistinguishable from that observed in BALB/c control mice (Fig. 6), indicating that *nmf375* mice are also resistant to changes in hippocampal synaptic plasticity due to USP14 deficiency.

### Characterization of peripheral motor and sensory circuits in 10-12 week-old *nmf375* mice

**Figure 7:**
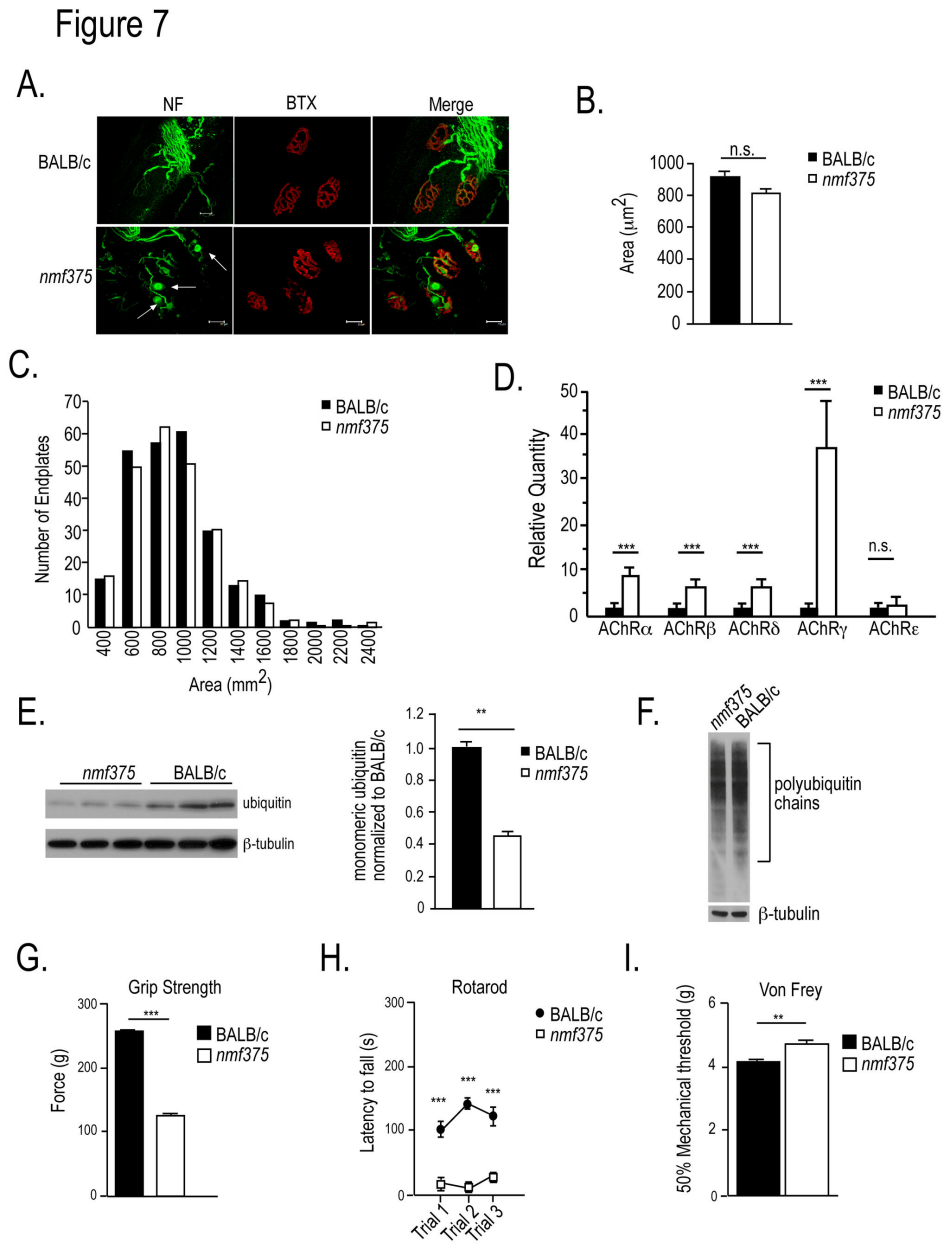
Analysis of NMJ structure, ubiquitin levels, and muscle AChR subunit expression in 10-12-week-old BALB/c and *nmf375* mice. *A*, Confocal images of whole-mount immunostaining of TA muscles stained with antibodies for neurofilament (green) and α-bungarotoxin (red). Terminals from *nmf375* mutants displayed accumulations of neurofilaments not observed in controls (white arrows). 63X magnification, scale bar 50 μm. *B*-*C*, Quantitation of the area (*B*) and size distribution (*C*) of the motor endplates. *D*, Quantitation of AChR subunit expression in gastrocnemius muscle (***p-value <0.001, n = 4). *E*, Immunoblot of ubiquitin from spinal cord extracts of BALB/c and *nmf375* mice. Quantitation of the immunoblot revealed a 55% reduction in monoubiquitin (**p < 0.01, n = 3). *F*, Representative immunoblot of total brain extracts from BALB/c and *nmf375* mice probed for polyubiquitin conjugates. *G*, Analysis of forelimb grip strength. *H*, Rotarod analysis ***I***, von Frey analysis of tactile sensitivity. For ***G-I***, n = 4-6 mice per genotype. *p-value <0.05, **p-value <0.01, ***p-value <0.001. Data were analyzed by Student’s t-test and are shown as mean ±SEM.

 Although USP14 deficiency in *nmf375* and *ax* ^*J*^ mice leads to motor deficits early in development, the neuromuscular phenotype is much less severe in the *nmf375* mice and does not appear to correlate with endplate pathology at 4-6 weeks of age. Since loss of UCH-L1, another DUB that is responsible for ubiquitin stability, leads to adult-onset endplate pathology, and the *nmf375* mice have an extend lifespan (Fig. 1E), we next examined if the *nmf375* mice exhibited adult-onset motor endplate disease. When we performed whole-mount immunostaining of TA muscles from 10-12 week-old *nmf375* mice (Fig. 7A), we did not observe any alterations in the endplate area (Fig. 7B) or size distribution of NMJ endplates (Fig. 7C) between the *nmf375* mutants and controls. However, the NMJs from the 10-12 week-old *nmf375* mutants had large accumulations of neurofilaments at the nerve terminals, which were similar to terminals of 4-6 week-old *ax* ^*J*^ mice (Fig. 7A) [16]. With the except in the AChRε subunit, examination of AChR subunit expression in these older *nmf375* mice also revealed an even greater upregulation in all subunits compared to what was observed in 4-6 week-old *nmf375* mice (Fig. 7D). When we examined motor performance in the 10-12 week-old *nmf375* mice, there was a significant reduction in the *nmf375* mutant as compared to controls (Fig. 7G and H). The 10-12 week-old *nmf375* mutants also displayed a similar deficit in tactile sensitivity to that seen in the 4-6 week-old *nmf375* mice (Fig. 7I). This result provides evidence that USP14 is required for maintenance and function of adult motor and sensory circuits.

We have previously shown that USP14’s role in maintaining ubiquitin pools is critical for the proper structural and functional development of the NMJ [16–18]. To determine if a failure to maintain ubiquitin pools with age contributes to the deficits observed in the older *nmf375* mice, we measured monomeric ubiquitin levels in 10-12 week-old mice. There was a 55% reduction in the levels of monomeric ubiquitin in *nmf375* mutants as compared to controls (Fig. 7E), providing further evidence that the NMJ pathology observed in USP14-deficient mice is linked to ubiquitin depletion. There were no detectable changes in the levels of polyubiquitin conjugates in the *nmf375* mice as compared to controls (Figure 7F).

### Genetic background alters disease phenotypes in USP14 deficient mice

**Figure 8:**
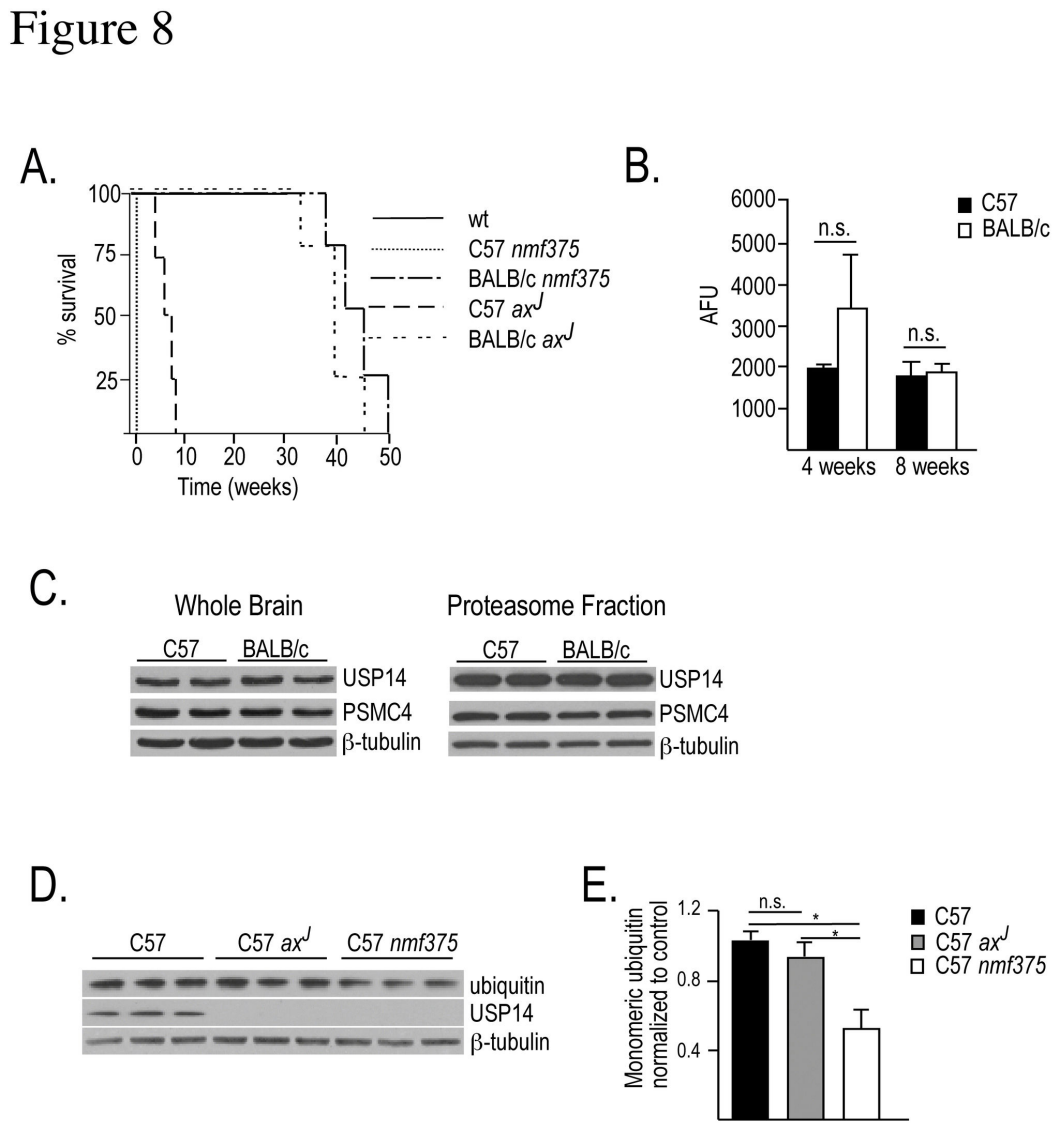
Analysis of *nmf375* and *ax* ^*J*^ mutations on C57BL/6J and BALB/c backgrounds. *A*, Survival curves for wt (C57BL/6J and BALB/c), BALB*ax* ^J^ and C57*nmf375*. N=4 mice per genotype. *B*, The trypsin-like activity of the 20S proteasome in arbitrary fluorescence units (AFU) from BALB/c and C57 mice was examined at 4 and 8 weeks of age (n = 2-4 per genotype, per age; p = 0.15 and 0.80, respectively). *C*, Western blot of proteasomal subunits in whole brain and proteasomal fractions from BALB/c and C57 mice (n = 2-3 per genotype). *D*, Representative western blot of monomeric ubiquitin in whole brain extracts from embryonic day 17-E18 C57, C57*ax* ^*J*^, and C57*nmf375* mice (n = 3 per genotype). *E*, Quantitation of monomeric ubiquitin levels (2-way ANOVA; *p-value < 0.05). Data were analyzed by Student’s t-test and are shown as mean ±SEM.

 One fundamental difference between the *ax* ^*J*^ and *nmf375* mice is the genetic background on which each mutation is maintained. The *ax* ^*J*^ mutation is maintained on a C57BL/6J background, while the *nmf375* mutation is on a BALB/c background. Because genetic background can have a major effect on disease onset and progression [30–32], we examined the role of genetic background on the phenotypic differences observed in our two USP14-deficient models. We first bred the *nmf375* mutation onto the C57BL/6J background for 10 generations and observed the phenotype of the resulting offspring. Surprisingly, on this genetic background, all *nmf375* mutants died at or before birth, suggesting that the *nmf375* mutation has a greater effect on *Usp14* expression than the *ax* ^*J*^ mutation (Fig. 8A). Consistent with this idea, knockout of the USP14 gene on a C57BL/6J background results in embryonic lethality by embryonic day 14 [37]. A single gene or multiple genes in the BALB/c background may therefore be capable of suppressing the developmental deficits caused by the loss of USP14. To test this idea, we bred the *ax* ^*J*^ mutation onto the BALB/c background for 10 generations and examined the phenotypic expression of the homozygous mutants (Figure 8A). On this genetic background, homozygous *ax* ^*J*^ mutants lived to at least 7 months of age, and displayed an adult-onset motor phenotype similar to what we have described for the *nmf375* mutants on the BALB/c background. 

Since the degradation of ubiquitinated substrates by the proteasome is significantly increased in the absence of USP14 [33,34], and may contribute to the phenotypes observed in the USP14 deficient mice [35,36], a reduction in 20S proteasome activity may provide a mechanism for suppressing the USP14 deficiency in the BALB/C mice. We therefore sought to determine whether alterations in 20S proteasome function could contribute to the differences observed between genetic backgrounds. To accomplish this, we measured the levels of proteasomal proteins and proteasome activity in BALB/c and C57BL/6J mice. No differences were detected in either proteasome activity (Fig. 8B) or protein abundance (Fig. 8C) between backgrounds. Collectively, these data support the observation that the C57BL/6J background is more susceptible to the loss of USP14 than the BALB/c background, and the resilience of the BALB/c background to USP14-deficiency is not likely to be due to differences in proteasome function between the two strains. 

Because the severity of the neuromuscular phenotype correlates with depletion of monomeric ubiquitin, we hypothesized that ubiquitin depletion underlies the neonatal lethality observed in C57*nmf375* mice. When whole brain extracts from C57, C57*nmf375*, and C57*ax* ^*J*^ mice at embryonic day 17-18 were immunoblotted for monomeric ubiquitin, no differences were detected in the levels of ubiquitin between the C57 controls and the C57*ax* ^*J*^ mice (Figure 8D). However, the C57*nmf375* mice had a significant reduction in ubiquitin compared to controls (Figure 8D). 

## Discussion

We have identified an ENU-induced, recessive mutation that presents with progressive muscle weakness, adult-onset neuromuscular disease, and premature lethality by 50 weeks of age. The *nmf375* mutation mapped to a region that contained *Usp14*, a gene previously characterized by our laboratory through studies of the hypomorphic, *ax* ^*J*^ neurodevelopmental mutation that arose on the C57BL/6J strain. The *ax* ^*J*^ mutant has prominent peripheral deficits at an early onset with a tremor, growth retardation, and slow, unsteady gait at weaning. Reduced muscle development and rigidity restricts the mobility of the *ax* ^*J*^ mice by 5-6 weeks of age and leads to premature lethality around 8 weeks. We found that the *nmf375* mutation is allelic and non-complementing to the *ax* ^*J*^ mutation. Although the C57*ax* ^*J*^ and BALB*nmf375* mutants appear to have similar loss of USP14, the difference between the phenotype of these mutants at 4-6 weeks of age is striking. While BALB*nmf375* mutants are able to perform similarly to controls in the open field task, C57*ax* ^*J*^ mutants are immobile. In addition to advanced mobility, the lifespan of BALB*nmf375* mutants is 6-fold greater than C57*ax* ^*J*^ mutants. The identification of the BALB*nmf375* mutant therefore yields a unique opportunity to investigate the role of USP14 in the maintenance of the adult peripheral nervous system. 

While the BALB*nmf375* mice were able to avoid the more extreme phenotypes of the C57*ax* ^*J*^ mice, loss of USP14 in the BALB*nmf375* mice was not without consequence. We found that BALB*nmf375* mutants had milder motor and sensory deficits at 4-6 weeks of age, reaffirming the role of USP14 in the development of the peripheral nervous system. The BALB*nmf375* mutants exhibited reduced grip strength and impaired motor coordination, as tested by the grip strength and rotarod assays. Utilizing von Frey analysis, we established a previously uncharacterized role for USP14 in tactile sensation; BALB*nmf375* mutants were hyposensitive to tactile stimulation compared to controls at 4-6 weeks of age. Analysis of *Usp14* expression during development demonstrated robust expression in the dorsal root ganglia [38], which was consistent with altered sensory function in the BALB*nmf375* mice. Our previous studies were unable to explicitly test the *ax* ^*J*^ mice for sensory deficits due to the presence of severe tremors and motor deficits. 

*Usp14* encodes a member of the ubiquitin-specific protease family of DUBs. USP14 associates with the proteasome and trims ubiquitin from proteins that have been targeted for degradation, serving to maintain ubiquitin pools within the cell [16–18]. Loss-of-function mutations within *Usp14* lead to a reduction of monomeric ubiquitin in both neuronal and non-neuronal tissues [18]. We have previously established that transgenic expression of ubiquitin in the nervous system of the *ax* ^*J*^ mice rescues the developmental and functional deficits caused by loss of USP14, demonstrating the critical role of USP14’s deubiquitinating activity in the maintenance of ubiquitin pools within the nervous system [16,17,39]. Although we were able to detect a significant decrease in monomeric ubiquitin levels in adult BALB*nmf375* mice, we did not find any detectable changes in ubiquitin levels in the 4-6 week old BALB*nmf375* mice even though they showed deficits in peripheral nerve function. Analysis of the ubiquitin transcripts *Ubb* and *Ubc* at 4-6 weeks of age demonstrated a significant upregulation of both transcripts in the BALB*nmf375* mice, suggesting that ubiquitin protein levels were maintained by increased transcription of the ubiquitin genes. Since *Ubb* and *Ubc* are the major ubiquitin producing genes and are normally down-regulated in adult mice, the decrease in steady state levels of ubiquitin only appear to become significant in the BALB*nmf375* mice when ubiquitin synthesis was limiting.

Our studies on NMJ structure in the *nmf375* mutants provide further evidence for an essential role for USP14 at the NMJ. Our previous studies with the 4-6 week old C57*ax* ^*J*^ mice demonstrated that functional deficits of synaptic transmission at the NMJ occur simultaneously with neurofilament accumulation, sprouting, and terminal swellings. In contrast, 4-6 week old BALB*nmf375* mice had no significant alterations in the structure of the NMJ compared to controls, but instead showed significant increases in AChR subunit expression, suggesting that the altered synaptic transmission was not dependent on altered NMJ structure. However, the BALB*nmf375* mice developed a pronounced motor endplate disease as they aged, which correlated with a significant loss of ubiquitin. These results suggest that the ubiquitin recycling activity of USP14 is required to maintain the structural integrity at NMJs, but an additional function for USP14 may also be required to maintain synaptic transmission.

Using the BALB*nmf375* mice, we have provided data establishing a critical role for USP14 in peripheral nervous system maintenance in adult mice. Our previous studies have demonstrated that USP14 is also required to regulate synaptic function in the hippocampus. Loss of USP14 in the *ax* ^*J*^ mice results in a decrease in presynaptic function as measured by PPF [14]. To determine whether the BALB*nmf375* mice were also resistant to the PPF deficit caused by USP14 deficiency in the C57*ax* ^*J*^ mice, we analyzed PPF in the CA1 region of the *nmf375* hippocampus. We found that the BALB*nmf375* mutants did not have a PPF deficit at 4-6 weeks of age, suggesting that the central nervous system of BALB*nmf375* mice is unaffected by the loss of USP14.

Our studies indicated that the genetic background of the mice contributed to the differences observed between the BALB*nmf375* and C57*ax* ^*J*^ phenotypes. To test this hypothesis, we crossed the mutations onto the opposite backgrounds for 10 generations. The *nmf375* mutation on the C57BL/6J background led to an exaggerated phenotype with homozygous mutants dying shortly before or after birth. We also found a greater loss of ubiquitin at embryonic day 17-18 in C57*nmf375* mutants compared to C57*ax* ^*J*^ mutants. This result suggests that the *nmf375* allele is a more severe hypomorph than the *ax* ^*J*^ allele and may therefore produce less USP14. In contrast, moving the *ax* ^*J*^ mutation onto the BALB/c background resulted in adult-onset neuromuscular disease, similar to what was observed in the BALB*nmf375* mutants. 

We have provided data arguing for a developmental requirement of a critical level of monomeric ubiquitin within the nervous system. The characterization of the *nmf375* mutants revealed that the BALB/c background is capable of maintaining ubiquitin levels through perinatal development in the absence of USP14, which may prevent the more severe phenotypes observed with USP14 deficiency on the C57BL/6J background. However, ubiquitin levels are not maintained in adult BALB*nmf375* mice. In adulthood, we see a 55% decrease in monomeric ubiquitin in *nmf375* mutants compared to controls, which correlates with the onset of neuromuscular disease. This result suggests that USP14 has an essential function in maintaining ubiquitin pools in the adult peripheral nervous system. 

We speculate that the expression and/or activity of another DUB is enhancing the recycling efficiency of ubiquitin during neurodevelopment. Roughly 100 DUBs are estimated to exist in mammalian cells, only 3 of which have been thought to associate with the proteasome by traditional purification methods [13,40]. Affinity purification of synaptic proteasomes has recently shown that USP5, USP7 and USP13 may also associate with the proteasome, indicating that proteasome composition may vary in different cellular locations or developmental stages to support unique aspects of neuronal function [40]. It is quite possible that a change in the expression of one or more of these DUBs within the first two months of development protects the BALB*nmf375* synapses from disease by assisting in ubiquitin recycling. Alternatively, UCH-L1, another DUB that plays a role in ubiquitin homeostasis in neurons, could help to stabilize ubiquitin expression in the absence of USP14. In contrast to USP14, UCH-L1 is thought to prevent the inappropriate degradation of ubiquitin by sequestering monomeric ubiquitin [41]. However, UCH-L1 loss also causes adult-onset motor endplate disease in mice [42]. The UCH-L1 and USP14 mutant mice also exhibit similar alterations in NMJ synaptic transmission, suggesting that changes in ubiquitin homeostasis may underlie motor endplate disease in both of these mutant mice [16,42]. Therefore, multiple mechanisms appear to control ubiquitin stability at synapses, and future studies will investigate the contribution of these mechanisms to the maintenance of ubiquitin homeostasis in different genetic backgrounds.

We have identified the causative mutation of the ENU-induced *nmf375* mouse mutant as a splice site mutation within *Usp14*. Characterization of this mutation demonstrated that the *nmf375* allele is a severe hypomorph, even more so than the *ax* ^*J*^ allele. However, *nmf375* was generated on the BALB/c strain that our studies revealed contains a suppressing element(s) that masks the severity of the *nmf375* mutation by possibly stabilizing ubiquitin expression during neurodevelopment. In adulthood, *nmf375* mutants have a significant decrease in the level of free ubiquitin that correlates with the appearance of swollen motor endplates and progressive motor defects. We provide evidence that the ubiquitin depletion observed in BALB*nmf375* mutants is independent of proteasomal activity, demonstrating that USP14’s role in the maintenance of monomeric ubiquitin pools is critical to the development and maintenance of the peripheral nervous system. 
